# Current Status and Nutritional Value of Green Leaf Protein

**DOI:** 10.3390/nu15061327

**Published:** 2023-03-08

**Authors:** Connor Balfany, Janelle Gutierrez, Marvin Moncada, Slavko Komarnytsky

**Affiliations:** 1 Plants for Human Health Institute, NC State University, 600 Laureate Way, Kannapolis, NC 28081, USA; 2 Department of Food, Bioprocessing, and Nutrition Sciences, North Carolina State University, 400 Dan Allen Drive, Raleigh, NC 27695, USA

**Keywords:** green biomass, plant leaves, alternative proteins, amino acid, functionality, complete protein, nutritional value, leaf protein concentrate

## Abstract

Green leaf biomass is one of the largest underutilized sources of nutrients worldwide. Whether it is purposely cultivated (forage crops, duckweed) or upcycled as a waste stream from the mass-produced agricultural crops (discarded leaves, offcuts, tops, peels, or pulp), the green biomass can be established as a viable alternative source of plant proteins in food and feed processing formulations. Rubisco is a major component of all green leaves, comprising up to 50% of soluble leaf protein, and offers many advantageous functional features in terms of essential amino acid profile, reduced allergenicity, enhanced gelation, foaming, emulsification, and textural properties. Nutrient profiles of green leaf biomass differ considerably from those of plant seeds in protein quality, vitamin and mineral concentration, and omega 6/3 fatty acid profiles. Emerging technological improvements in processing fractions, protein quality, and organoleptic profiles will enhance the nutritional quality of green leaf proteins as well as address scaling and sustainability challenges associated with the growing global demand for high quality nutrition.

## 1. Introduction

Globalization is intertwined with socioeconomic growth, and the expanding populations in both emerging and advanced markets demand more protein that is affordable, sustainable, and nutritious [1]. Health attributes, with a particular focus on increased consumption of plant-based proteins, are rapidly becoming major product traits sought by consumers [2]. To stay ahead in the food value chain, the industry movement toward increased protein production is gradually matched with consumer demands. Among the newly advancing protein technologies, cultivated meats (animal cell culture), fermentation protein (bacterial, fungal, or algae biomass), precision fermentation (recombinant protein expression), novel animal sources (i.e., insect protein), and alternative plant proteins have recently reached either commercial or late research stages [3]. The global alternative protein market for animal feed, whether in the form of novel feed ingredients (i.e., oilcake proteins) or novel forage crops that bring additional agronomic or ecological value, is also expected to have a positive impact in all areas of modern food production. The conventional protein markets could be seriously disrupted by these technologies, especially as production prices drop. The stakes are high as global protein ingredient market was capitalized at USD 38B in 2019 and projected to grow 9% per year in the next 10 years [4].

Ongoing research, therefore, aims to identify “new” and reasonably priced sources of plant protein, including agricultural production waste streams in the form of fruit pomaces and distiller’s grains as well as discarded seeds, offcuts, peels, pulp, and green leaf biomass. Extracted plant proteins can be utilized in the form of flours (20%+), concentrates (70%+), isolates (90%+), or hydrolysates of various degree and may contain other plant-derived confounders such as fibers, starches, and bioactive phytochemicals that hold the potential to modify energy density and organoleptic properties of the final product. When produced at competitive prices, these proteins hold high potential to offset the market share from conventional animal and plant proteins (meat, dairy, eggs, and whole legumes). The rising popularity of vegan, vegetarian, and flexitarian diets provide additional support to this trend and creates a unique opportunity to develop and manufacture a wide range of novel protein products. Alternative plant protein ingredients may also provide additional functional features such as decreased allergenicity (when compared to dairy, eggs, and soy-based products), or additional clean label opportunities to achieve structure, stability, emulsification, and flavor enhancement goals.

The equivalency or superiority of novel alternative proteins or protein feed ingredients must be extensively demonstrated before they are expected to gain market success. This includes the development of efficient protein extraction and manufacturing strategies that ensure competitive high yields and enhanced preservation of functionality traits. Recent technological advances have begun to position plant proteins as viable alternatives to support stabilization (milk casein), viscosity (whey proteins), foaming networks (egg white proteins), and gelation (muscle myofibrillar, sarcoplasmic, and stromal proteins) goals critical to many food processing and manufacturing industries. The texture and flavor challenges must be overcome or utilized to their advantage in novel ingredients with enhanced health promoting or sensory profiles. This review looks into a number of underutilized plant protein sources, describes possible extraction strategies to enhance their yields and preserve functionality, and discusses factors that influence acceptance and demand for novel food protein ingredients with a particular focus on green leaf biomass.

## 2. Conventional Protein Sources and Their Alternatives

The recommended dietary allowance (RDA) of protein for a healthy adult is in the range of 0.8–1.6 g protein/kg body weight per day depending on the level of physical activity and rates in excess of 2 g/kg/day are not generally recommended [5]. Often overlooked is the fact that most of this protein globally comes from plants (57%), with meat (18%), dairy (10%), fish (6%), and other animal products trailing behind [6]. This ratio is skewed in favor of animal protein (55–60%) in Oceania, Europe, and Americas [7]. Animal proteins score higher on protein content, digestibility, net protein utilization, biological value, and on the protein digestibility-corrected amino acid score that accounts for human amino acid requirements (PDCAAS), and the digestible indispensable amino acid score that incorporates information on digestible amino acids and their ileal digestibility (DIAAS) (Table 1). This is explained in part by increased incidence of hydrophobic β-sheet protein structures, polysaccharide fibers, and antinutritive (or rather antidigestive) factors in plant tissues [8]. Additional treatments during the processing (soaking for phytic acid, heating for protease inhibitors and cyanogens, etc.) or the manufacturing of protein concentrates and isolates can improve their digestibility closer to that of the animal products [9]. Accurate flavor profiling of plant protein products during the manufacturing process may also allow for complete removal of the undesirable off-flavors in the future [10].

With consumer demand unlikely to fall, animal meat alternatives with improved undeniable environmental and animal welfare impacts will be essential. Several emerging technologies are positioned to become mainstream when price parity and nutritional parity are reached. While the former is projected to become possible within a few years, the latter is lagging behind due to the ultraprocessed nature of these products and will require an unconventional paradigm shift away from mimicking animal products (meat analogues) to creating its own category that can address the nutritional challenges of the current agricultural production systems [3]. The emerging animal-based and plant-based industries are also projected to generate a new series of waste streams that need to be recognized and utilized.

### 2.1. Cultivated Protein

The category of cultivated proteins encompasses predominantly cultivated or cultured meats; however, algal and insect protein cultivation can be loosely associated with this group as well (all these cultivation techniques require oxygenation). These proteins are produced with the major goal of achieving parity with expensive animal products, such as whole or minced meats, and with most efforts focused on the development of acceptable ingredients that mimic meat texture and flavor profiles. Achieving parity with eggs and dairy is not expected short term, as these products cost less and the key proteins responsible for their functionality can be successfully produced using various fermentation technologies as discussed below.

#### 2.1.1. Cellular Agriculture of Animal Protein

Cellular in vitro agriculture of animal muscle and fat tissues were envisioned as early as the 1930s and reduced to a routine practice in 2013. Significant investments from both industry and academic stakeholders led to rapid technological advances that improved product quality and accelerated the time to parity from 1.2 M USD/lb in 2013 to 50 USD/lb in 2021, although costs, cell sourcing and culture medium composition, scaleup (bioreactor capacity), and biological limitations of the animal cell culture systems remain a challenge [11]. The consumer appeal and private interest in the technology is substantial, as considerations for improved animal welfare [12] and environmental impact [13] continue to drive modern purchasing decisions in food products [14]. Policy surrounding cellular agriculture is also in its infancy, with regulations and legislation focusing more on labeling, branding, and food safety restrictions as consumer perception changes under the different names and marketing strategies [15]. Nutritional parity of cultured meats is difficult to achieve since conventional meats are enriched with often highly bioavailable minerals (iron, zinc, and selenium) and vitamins (A, B9, B12, D, and E) [16].

#### 2.1.2. Algal Bioreactors

Algae is a diverse polyphyletic group ranging from the largest single cell organism *Caulerpa taxifolia* (M.Vahl) C. Agardh to the largest cloned seagrass *Posidonia australis* Hook. F; however, algal protein manufacturing currently focuses on unicellular microalgae. Rapid doubling times as low as 1.5 h for chlorella and the naturally high protein content ranging from 40 to 60% are the key metrics in creating sustainable and highly efficient algal protein production systems [17]. Algal bioreactors show one of the lowest environmental footprints with an average of 2.5 m^2^ land area use per kg of protein produced, which compares favorably not only with animal production systems (42–258 m^2^), but also with grains and beans (22–46 m^2^) [18]. Algal protein is a complete protein with adequate amounts of every amino acid; however, aspartic and glutamic acids can constitute 22–44% of amino acids in some brown seaweed species, which could lead to systemic acidosis if consumed in high enough quantities [19], and its lower digestibility is critically dependent on the rigid properties of the microalgal cell wall [20]. Alginates in brown seaweed and carrageenan in red seaweed further decrease the efficiency of protein extraction [21]. Algal protein has a stronger flavor profile and is associated with higher energy costs due to the rigidity of algal cell walls [22]. These combined technological challenges are, therefore, the major reasons why most of the algal biomass production is currently geared towards animal feeds [23].

#### 2.1.3. Insect Protein Farms

Insect protein has served and continues to serve a large role in many nonwestern diets, and rejection of insect-based foods is largely a learned behavior [24]. The insect protein production industry has a unique opportunity to circumvent this pushback by producing the majority of insect protein for animal feed while developing advanced technological solutions to use insects in human food production [25]. Depending on the production species, both high value food sources and biowaste or agricultural waste streams can be used for rearing [26]. Insect protein is complete and ranges from 35 to 60% dry weight or 10 to 25% fresh weight [27]; however, research into its production, processing, and application to food matrices to achieve parity with other alternative proteins is critically lacking. Protein production by insect cells and their potential application in large-scale manufacturing faces the same challenges and bottlenecks that apply to animal cells even though insect cell lines with high protein yield and greater passaging stability are readily available [28].

### 2.2. Fermentation Protein (Traditional, Biomass, and Precision)

Microbial fermentation cultures are able to produce protein products in the absence of oxygen. Traditionally used in the preservation of animal (cheese, yoghurt, kefir) and plant (tempeh, tofu, sauerkraut, wine) foods [29], fermentation technologies can be extended to target total protein biomass (marmite, mycoprotein) [30] or a particular protein based on the synthetic DNA expression in the host microbial cells [31]. Fermentation also has the capacity to improve incomplete protein profiles of fermentation substrates and achieve high 40–75% dry weight protein yields [32].

Recently, many industry players focused their efforts on advancing fermentation technologies to produce bulk protein (Quorn Foods), or functional proteins such as casein (Perfect Day), leghemoglobin (Impossible Foods), egg white (Every Food), or collagen (Geltor). Strain screening, early-stage bioprocess development, growth optimization, and substrate selection are critical for achieving commercial grade biomass yield and productivity [33]. Fermentation is one of the most interesting alternative protein markets within the agricultural food systems to watch and the one with higher chances to achieve price parity with conventional meats in the short term.

## 3. Plant-Based Alternatives and Undervaluation of Green Leaf Biomass

**Figure 1:**
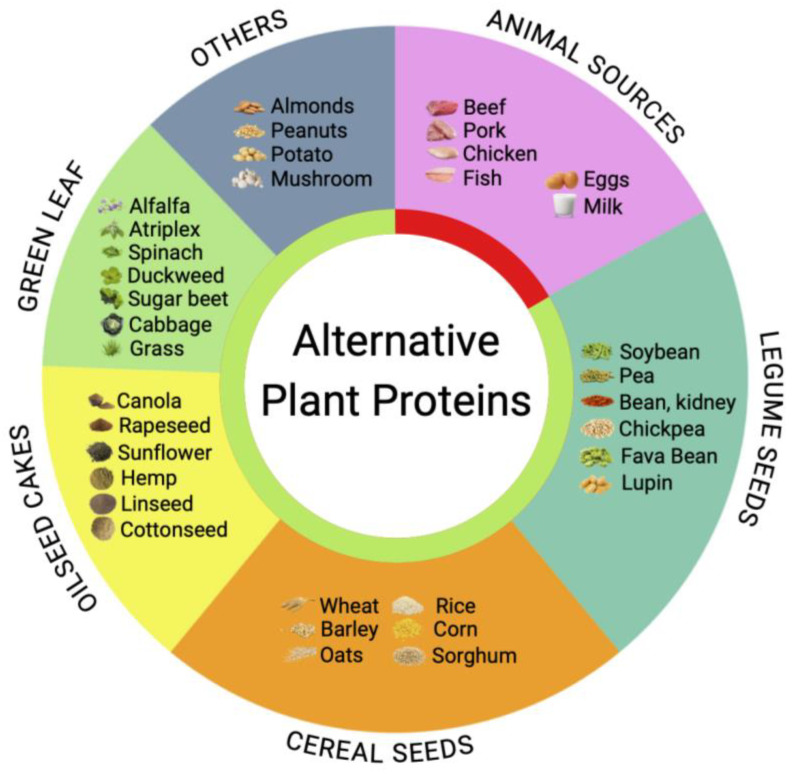
Major groups of alternative plant-based proteins (green) contrasted with the traditional proteins from animal sources (red).

Vegetal sources of protein dominate the global protein supply and account for as much as 60% of protein provided [6]. However, plant proteins may not contain all the essential amino acids in the required proportions (Table 1). The bulk of this protein comes from consumption of plant seeds as a part of the omnivorous human diet. Seed storage proteins naturally accumulate in the cotyledons and embryos of dicots (i.e., pulses) or the endosperm of monocots (i.e., cereals). These can be mechanically fractionated and, therefore, enriched for downstream protein processing using milling, air classification, and steeping [34]. In this group, soy, pea, chickpea, and bean proteins are the most widely used. Additionally, oilseeds provide protein-enriched press cakes (meals) following the initial dehulling and extraction of vegetable oils [35]. These waste products can be upscaled to produce bulk protein as shown for rapeseed, sunflower, and hemp crops, among others (Figure 1). Green biomass or green leaf protein remains a largely unexplored option in this landscape. Traditionally, these materials are used in direct foraging by ruminants or as a part of the animal feeds. Novel forage crops with improved protein and essential amino acid profiles suited for marginal or environmentally challenged soils as well as novel byproduct fractions produced from the green leaf biomass with improved digestibility or nutritional quality profiles represent an untapped opportunity to provide additional sustainable sources of plant proteins for human and animal diets.

### 3.1. Green Leaf Protein, Brief History and Past Uses

Anthropoids include at least some plant foliage in the diet by balancing intake of scarce, higher quality (low fiber, higher carbohydrate) fruits with abundant, lower quality (high fiber, higher protein) leaves, and this process seems to be reinforced by an active selection for soluble protein [36]. Historical consumption of plant leaves also explains the evolutional diversity of the human TAS2R bitter taste receptors [37] as well as the emergence of the umami TAS1R1/TAS1R3 heterodimer receptor highly and specifically sensitive to L-glutamate as an indicator of proteinogenic leaf substrates [38]. Green leaves are nutrient-rich foods that have been an important part of the traditional human diet, and their potential contribution to protein intake is often overlooked.

The first isolation of green leaf proteins was achieved by Rouelle in 1773 in the form of “*matiere glutineuse ou vegeto-animale*” with a burned feathers smell (proteins were not discovered or named until 1838). The method used involved many of the modern concepts of protein isolation and included pulping of the leaves, straining the juice, heating to obtain a green coagulum, and decolorizing it with alcohol. A similarity to the coagulation of egg whites and blood was proposed in 1792 by Beddoes, with possible application to human nutrition, but was supposedly ridiculed. Alkali extraction of dried and ground leaves was pioneered by Winterstein in 1901 and Osborne in 1920, while the optimized leaf protein extraction technology using heat precipitation was disclosed by Ereky in 1927 [39]. The early commercial application of green leaf protein was achieved in the form of the PRO-XAN protein-xanthophyll concentrate from alfalfa *Medicago sativa* L., developed as poultry feed, with the green fiber fraction used for ruminant feed [40]. The protein concentrate from the mixed grasses and berseem clover *Trifolium alexandrinum* L. was fed to 100 children for 8 months and shown to be comparable to milk protein supplementation [41]. These studies indicated proteins from green biomass as a viable alternative to soy and other seed proteins for food and feed applications (Figure 1).

### 3.2. Composition of Green Leaf Proteins

A large-scale screening effort to evaluate the green leaf protein content of 500 plants species harvested prior to flowering was undertaken by the USDA Tropical Agriculture Research Station in Puerto Rico in the 1970s. This study reported the green leaf dry matter in the range of 10.2–34.9% and the crude protein content at 10.8–35.7% dry weight [42]. When crudely processed, green leaves produce 50% leaf liquid (95% moisture content), 45% fiber (55% moisture), and 5% leaf protein concentrate (55% moisture) [43]. As such, the amount of protein in green leaves varies between 1.2 and 8.2% fresh weight depending on the species and cultivation settings, and in many plants, compares favorably to that of milk (3.5%), even when extracted at 50% efficiency (Table 1).

Depending on the plant species, the green leaf protein can be further fractionated (see Section 3.3). While plants are very diverse, they all share a common set of proteins in their photosynthetic tissues that enables the capture of sunlight energy and its use in carbohydrate synthesis [44]. Because of this, 75–80% of C3 plants’ total nitrogen is found in the green chloroplasts, with the CO_2_-fixing protein Rubisco accounting for 10–30% of total leaf nitrogen and up to 50% of soluble leaf protein [45]. Rubisco is a complex but very conservative protein consisting of 8 small and 8 large subunits (12.5 and 55 kDa, respectively) that, in a model spinach *Spinacia oleracea* L. plant, is characterized by the isoelectric point of pH 6.0–6.1 and the denaturation temperature of ~65 C [46]. Rubisco has advantageous functional features in terms of enhanced gelation, foaming, emulsification, and textural properties [47]. During the leaf protein fractionation, Rubisco, together with the other soluble proteins, forms a more desirable beige fraction of the leaf protein (white protein) [48].

The bulk of the remaining proteins are hydrophobic in neutral solutions and include cell wall proteins, cell membrane proteins, leaf storage proteins, and lectins. Many of these proteins have an increased capacity to bind polyphenols and fibrous polysaccharides due to their structural integration into cell walls and membranes, associate with thylakoid membranes of plant chloroplasts, and precipitate together with chloroplast fragments at a lower coagulation temperature of ~55 C, thus forming a less desirable green fraction of leaf protein (green protein) [49]. Additionally, selected plant species produce a minor fraction of tan protein at a higher coagulation temperature of ~82 C or show amorphous coagulation [42].

### 3.3. Extraction and Concentration

Crude isolation of green leaf proteins is achieved by pulping the leaves, pressing the pulp to obtain green leaf juice, then quickly bringing the green liquid to the boiling point to coagulate the leaf proteins and to partially pasteurize the final product. When starting with 2 kg of fresh green leaves, the process produces ~100 g of filtered green curd that contains ~50 g of crude protein and generates 1 kg of wet fiber and 1 L of liquid as byproducts [43]. The green leaf protein concentrate (LPC) can be used in animal feed as a part of the biomass utilization biorefinery setup [50]; however, the lack of many organoleptic and functional properties prevents its wider use in food product manufacturing.

In contrast with pulse, cereal, or oilseed protein extraction technologies, the mechanical separation and concentration steps of dry materials to achieve a protein-rich fraction are not applicable to green plant biomass. For this reason, in-depth studies into further fractionation and isolation of green leaf proteins focused on a small number of green crops such as alfalfa (lucerne), sweet potato *Ipomoea batatas* (L.) Lam. [51], or sugar beets *Beta vulgaris* L. [52] and narrowed down the juicing technology to the use of twin-screw extrusion as one of the most effective strategies [53]. Several downstream technological solutions exist to increase the yield and functionality of leaf proteins, albeit none of them are capable of addressing both issues simultaneously (Supplementary Table S1).

#### 3.3.1. Thermal-Assisted Extraction

Heat and pressure could be applied to the plant biomass to increase green juice recovery from spinach; however, it is associated with partial denaturation of plant proteins and functionality loss [54]. This application, however, could be used for a partial in-tissue precipitation of the green protein to assist with fractionation of the white protein.

#### 3.3.2. Alkaline Extraction

Alkali extraction solvents are used to increase recovery of proteins from the plant biomass of many established agricultural crops, including soybeans *Glycine max* (L.) Merr. [55], peas *Pisum sativum* L. [56], and barley *Hordeum vulgare* L. [57], among many others. High amounts of basic OH- ions partially degrade β-1-4 glycosidic linkages in cellulose and saponify lipids within the cell membrane, thus aiding in cellular disruption and increasing protein recovery [58]. Basic pH also disrupts disulfide bonds and increases protein solubility. This is achieved by adding sodium hydroxide, potassium hydroxide, calcium hydroxide or ammonia, although the latter requires an additional stripping column and, therefore, is more expensive [59]. Alkalization at lower temperatures decreases solubility but improves protein structure and functionality [60]. Dry and processed leaf tissue can be also extracted with alkali treatments, yet at lower efficiency [61]. However, basic extractions may lower the overall quality and applicability of the isolated protein by decreasing its lysine and cysteine content [58].

#### 3.3.3. Enzyme-Assisted Extraction

External enzymes can be used to aid in the degradation of cell wall components (carbohydrolases and cellulases that target hemicellulose, cellulose, or pectin) and enhanced the release of plant proteins, alone or in combination with other mechanical pretreatments [62]. Although added in relatively small amounts (0.2–5%), they change the economics of the protein extraction process due to the large volumes of treated biomass [58] and additional control of pH ranges [63]. These may also include proteases to enhance fractionation (hydrolysis) of high molecular weight proteins, increase solubility, and alter the final functionality of the resulting protein hydrolysates [64]. Protease mixtures may also aid in a partial breakdown of native protein complexes with carbohydrates, phytates, and chlorophylls [65] and may be more important in this process than carbohydrases [66]. The drawback of uncontrolled proteolytic hydrolysis is the generation of low molecular weight peptides and newly exposed hydrophobic amino acid clusters that results in increased bitterness and limited protein applicability.

#### 3.3.4. Ultrasound-Assisted Extraction (Sonication)

Liquified green biomass can also be subjected to ultrasonic treatment to aid in cellular disruption and protein release [67]. This treatment is often combined with alkaline and enzymatic-assisted extraction to allow for more extensive penetration and increased surface area as well as to reduce undesired enzymatic activity due to the direct inactivation of enzymes [68]. Technical challenges related to the uneven distribution of energy and the high costs of operation currently limit the commercial application of this approach, although recent studies with cauliflower byproducts [69] and blanched alfalfa [70] warrant further investigation on the subject.

#### 3.3.5. Pulse Electric Field-Assisted Extraction (PEF, Electroporation)

The permeability of plant biomass can be increased by rapid and repeated exposure to electrical pulses that cause partial disruption of cell walls and pore formation in cell membranes [71]. Similarly to ultrasound, PEF treatment can aid in the inactivation of enzymes [72] and increase protein yields as shown for alfalfa [73], albeit at a lower efficiency.

#### 3.3.6. Heat Precipitation

**Figure 2:**
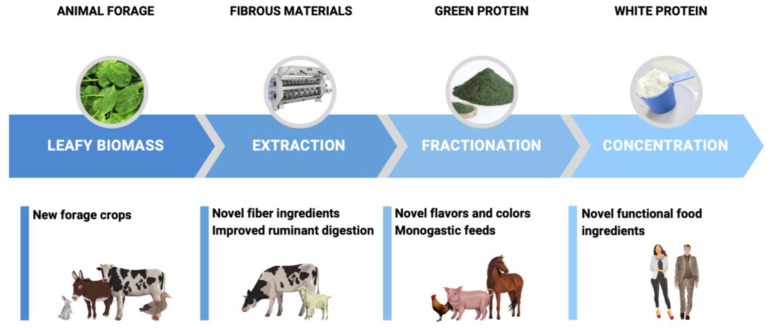
Schematic processing of green leaf biomass into various fractions with different applications in animal forage, ruminant animal feeds, monogastric animal feeds, and human food systems.

Green leaf protein must be concentrated before it can be successfully used in food manufacturing, and thermally induced precipitation continues to be a cost-effective method in modern day recovery systems by using heat exchangers or steam injectors. Due to inherent differences in denaturation temperatures, green proteins predominantly associated with membranes and chloroplasts can be separated from white (beige) soluble proteins in a two-step heat treatment at ~55 °C and ~65 °C, although exact temperatures must be established anew for each green biomass and manufacturing process [42] (Figure 2). Heat-coagulated protein, however, comes with a relatively high energy consumption and lower water solubility that hampers its application in food systems [74].

#### 3.3.7. Acid Precipitation (Isoelectric)

Alternatively, isoelectric precipitation by adjusting plant juice within the pH 3.0–5.0 range to precipitate proteins with hydrochloric acid [75], anaerobic fermentation including lacto-fermentation [76], or as a part of the cellulosic ethanol production process [61] may result in higher quality protein ingredients, especially when the pelleted proteins are neutralized before use [77]. This process takes advantage of the proteins aggregating at their isoelectric points. This is equally applicable to seeds, as shown for peas [56], and both high moisture (i.e., spinach) [78] and low moisture (i.e., cassava) [79] green leaves. Moreover, this process can be combined with prior thermal precipitation of green protein (wet fractionation) to obtain a more functional white protein enriched with the globulin fraction [80]. Variations of this process allowed for the initial attempts at commercial scalability of green leaf proteins with sugar beet tops (Cosun Beet, Roosendaal, The Netherlands), cabbage trimmings (Naylor Farms, Spalding, UK), and duckweed (Plantible Foods, Eldorado, CA, USA).

#### 3.3.8. Ultrafiltration

Ultrafiltration or diafiltration allow for more gentle recovery of the target proteins with enhanced functionality and for the enrichment of both globulin and albumin fractions of plant proteins [81]. This step can be also applied to proteins that were salted out from solution without heating or pH change [82]. This allows for a higher solubility, emulsification, and foaming capacity of the resulting ingredients, yet comes with higher costs due to membrane manufacturing and fouling [83].

### 3.4. Scaleup and Technological Concerns

The economics of extracting green leaf biomass for protein production was reviewed several times in the 1980s, 2010s, and as recently as 2021 [42,59,84,85]. As many green leaf protein extraction techniques were validated only at the lab scale, they lack the cost analysis required to implement them commercially. Wet fractionation (alkaline extraction followed by isoelectric precipitation) was estimated to recover 85% of proteins with a purity of 52% and to cost EUR 0.102 per kg of green protein in 2014 [60]. The economic feasibility of green protein or combined (green and white) protein was based on the breakeven price of EUR 2 per kg of bulk ingredients in 2021 when accounting for agricultural production and fertilization [84]. More expensive fractions may be justified based on the functionality profile and market application, as seen in whey protein and its application in sport nutrition [83].

The agricultural aspect of green biomass protein production is often overlooked and will likely require a vertical integration of a single agricultural entity that ensures crop selection, coordination of harvesting and production, processing of green biomass adjacent to crop land to minimize the transport and storage, and integration of livestock to capture fibrous materials and green protein (Figure 2). This may require development of novel agricultural food production systems based on partially controlled environment agriculture [3]. Duckweed, due to its unique nature of cultivation, may offer some logistical advantages to this approach [86]. Target crops production should also be optimized for leaf rather than stem, and young rather than old leaf production, to limit processing of more fibrous plant tissues. Focus on the utilization of agricultural byproducts of existing crops available year-round (fruit pomaces, distiller’s grains, discarded seeds, offcuts, peels, pulp, or tops) may offer additional advantages. For processes that include spent green biomass such as tea leaves, a reversed biorefinery approach starting with ethanol-assisted extraction of phytochemicals, followed by mild (55 °C, pH 9–11) and severe (95 °C, pH 13) protein extraction, and an ultimate harsh hydrolysis to release monosugars (100 °C, 0.5 M alkali) may be considered [87].

## 4. Leaf Protein Quality and Nutritional Outcomes

Nutritional and functional profiles of green leaf proteins vary depending on the source of the protein (Table 1), the degree of purification, and the extraction strategy used. Likewise, the different extraction steps may be used to intentionally modify the physicochemical and nutritional properties of these alternative proteins. Additional processing steps that may influence the protein profile include the use of organic solvents (ethanol, acetone, 1-butanol) to remove chlorophyll and phytochemicals [88], flocculants that may remain in the final product [89], spray drying that may concentrate minerals and salts in the protein powder [90], and the duration of treatment that may increase endogenous proteolysis and oxidation [91]. This intrinsic complexity of green leaf protein ingredients does not allow for a straightforward comparison of their functional and nutritional values among different agricultural crops and their byproducts. The following sections will, therefore, briefly summarize the key factors critical for evaluation of protein ingredients as they apply to green leaf proteins, while the comprehensive summaries can be found in other recent reviews [9,92].

### 4.1. Functionality of Protein Ingredients

Structural properties of the food matrix define its palatability, texture, digestibility, bioavailability of nutrients, and the shelf life of the final product [93]. Protein ingredients are inherent food structures that have the capacity to form gels that entrap liquid, stabilize emulsions and enhance the encapsulation of oils, increase stability of foams, and modulate texture (hardiness, mouthfeel) [94]. These properties are largely defined by the amino acid composition, the presence of intermolecular bonds, and the structural confirmation of the protein [95,96].

#### 4.1.1. Solubility

The solubility of a protein is the most important determinant in its application to food systems, as it directly impacts protein–protein interactions and modulates the majority of the functional outcomes discussed in this section. The overall charge of the protein defines its solubility in aqueous solutions of various pH and ionic strength. The solubility of green leaf proteins is at minimum in the pH range close to the isoelectric point of Rubisco (pH of 3.5–5.0), slightly increases at pH 2, and reaches maximum solubility at pH 9–11 [52]. The majority of luminal and cytosolic leaf proteins are highly soluble, while the proteins associated with thylakoid and plasma membranes show decreased solubility [86]. This can be partially improved by a successful pH-shift approach that solubilizes proteins at alkaline pH 11–12 with chemical additives such as NaOH and NH_4_OH before neutralizing the protein solution at pH 7 with HCl [97]. Protein solubility can be further improved with high pressure homogenization [98], glycation-induced structural modification [99], ultrasonic treatment [100], or enzymatic hydrolysis [101].

#### 4.1.2. Gelation

The partial unfolding of protein structures to allow for the formation of intermolecular polymer bonds results in the establishment of elastic protein aggregate networks (gels) that capture liquids, dissolved food ingredients, and flavors. This is achieved by heating the soluble protein to a dissociation temperature of 70–90 °C and cold-setting [102]. Green leaf proteins form strong gels at relatively low concentrations (2–10%) that perform better than whey protein, soy protein, or egg white protein as shown for sugar beets [52], duckweed [86], or spinach [46]. Gelation can be partially improved by the high temperature, short time extrusion of the protein ingredients [103], or enzymatic treatment with transglutaminase [104].

#### 4.1.3. Foaming and Stability

The foaming of the protein depends on its ability to position itself at the air–solution interface and to maintain this configuration. This is achieved through interactions of polar and hydrophobic amino acid regions with the respective media and also defines the water and oil absorption capacity of the protein, respectively. Green leaf proteins form better foams at lower pH 2–3 [105] than neutral pH 7 [52]. Foaming and absorption capacities of protein ingredients may be improved by homogenization [106] and acetylation [107], while hydrolyzation is generally detrimental [108].

#### 4.1.4. Emulsifying Properties

A good balance of polar and hydrophobic regions is also required for the protein to interact well with both water and oil to form emulsions. This can be achieved by partial denaturation of the proteins to expose the hydrophobic regions; however, the degree of denaturation that produces the smaller and more desirable micelle size is specific to the target protein ingredient and difficult to control [109]. While certain plant proteins are good emulsifiers as shown for soy [110] and potato [111], the emulsifying properties of green leaf proteins are not well-studied. Recent studies indicated that Rubisco protein showed average emulsification properties [52], which seemed to improve with a more alkaline pH in case of alfalfa [112]. A partial denaturation increases emulsification when caused by high pressure homogenization [98], glycation [99], and a combination of pH shifting and ultrasonication [100].

### 4.2. Nutritional Aspects of Green Leaf Protein

Nutrient profiles of green leaf biomass (plant leaves) differ from those of plant seeds in protein quality, vitamin and mineral concentration, and omega 6/3 fatty acid profiles [113]. In contrast to cereal grain proteins deficient in lysine and/or tryptophan or legume seed proteins deficient in methionine and/or cysteine, green leaf proteins nearly match the FAO standard of a complete protein (Table 1). Green leaf concentrates (green crude preparation with approx. 50% protein content) are a good source of vitamins such as β-carotene (provitamin A), B6, B12, E, and K as well as several micronutrients including iron, calcium, and magnesium [43] that are regarded as common mineral inadequacies in modern diets [114]. In contrast to edible seeds enriched with omega 6 fatty acids, green leaves accumulate more omega 3 fatty acids including α-linolenic acid, a major precursor to the EPA and DHA metabolites [115]. Their nutritional value is, therefore, clearly on the side of increasing the proportion of green leaf biomass-derived food ingredients in the human diet, yet some challenges remain, as briefly described below.

#### 4.2.1. Amino acid Composition

When considering alternative proteins in human diets and animal feeds, aspects such as protein content, amino acid profile, digestibility, essential amino acid (EAA) deficiencies, antinutritional factors, and palatability must be addressed. Imbalances in essential amino acid (EAA) profiles are common for many plant proteins. They are defined by the indispensable amino acid content of a protein (mg/g) versus a theoretical reference (complete) protein, and the lowest ratio delimits the most limiting amino acid [116]. Legumes are often lacking in methionine, while grains are generally poor in lysine. Green leaf proteins satisfy the FAO standard of a complete protein similar to animal foods (Table 1). Their completeness is mostly defined by high Rubisco and other chloroplast protein content which is highly conserved at both gene and protein level [117]. When fractionated, Rubisco is expected to end up in the white fraction of green leaf proteins [42].

#### 4.2.2. Digestibility and Antinutritional Components

To further standardize the nutrient analysis of the protein, it is corrected for the fecal true digestibility (PDCAAS) or ileal digestibility (DIAAS). While most of the animal proteins have PDCAAS at or very near 1.0, plant protein scores are usually lower due to amounts of one or more indispensable amino acid and the presence of antidigestive (antinutritional) factors. In general, the digestibility of green leaves improves with processing, and is highest in the white fraction [118], while higher temperatures used during the concentration, isolation, or drying steps decrease leaf protein digestibility [119].

Green leaves are also a diverse source of secondary metabolites and other antinutritional (yet possibly health-promoting) [3] factors that may decrease protein digestibility, biological value, and net utilization by various mechanisms such as cross-linking and reduced solubility. These mechanisms include direct oxidation and formation of quinone complexes and dark melanin pigments mediated by the chloroplast-derived polyphenol oxidase, peroxidases, or laccase enzymes [120]. The resulting protein–phenol complexes showed a marked decrease in free amino groups, increased protein derivatization that reached maximum at pH 10, and changes in protein digestibility that inhibited pepsin and favored trypsin digestion [121]. This can be partially prevented with the addition of higher amounts of sulfites [122] or several alternative treatments [72]. Among the other secondary plant metabolites of concern, phytates generally do not accumulate in green leaves; however, tannins, saponins, lectins, chlorophyllides, protease inhibitors, oxalates, and phytoestrogens may decrease the quality of leaf protein concentrates and need to be addressed in a species-specific approach. While these purported antinutrients may decrease protein digestibility and absorption of certain minerals, recent findings in their application to other human health outcomes and metabolism by the human microbiome may warrant a re-evaluation of current guidelines on their safety and use in foods [123].

#### 4.2.3. Applications to Feed and Foraging Systems

Current conversion rates of plant to animal protein in the conventional agricultural food systems are estimated at only 3–13% [124]. Therefore, reallocating the agricultural land used for beef feed to production of alternative feeds may be environmentally sound and economically feasible. Additionally, focusing on green leaf crops that can be produced in the extreme or marginal ecological niches not utilized by current agricultural practices due to temperature, precipitation, or salinity may allow for the generation of additional revenue streams. Together, these factors hold the potential to disrupt the current economic cycle of using plant seeds as international commercial commodities, feeding seed protein to livestock, and using excess seed oils in the fast-food industry.

Marginal, dry, or high salinity pastures are limited in many nutrients, including vitamin E, resulting in nutritional myopathy and less hydrated carcasses. These can be partially compensated for with the introduction of alternative forages such as saltbush (*Atriplex cinerea* Poir.) [125] or orache (*Atriplex hortensis* L.) [126] as unexplored sources of green leaf protein. During the first step of green leaf protein isolation (Figure 2), near half of the protein with a comparable amino acid profile is captured in the insoluble green fibrous material (pulp) suitable for high quality ruminant feed production [127]. The use of novel forage crops and their green leaf proteins in animal feed can help to improve the nutritional value of the feed, enhance animal performance, and reduce reliance on traditional protein sources, especially on marginal soils and in areas affected by salinity.

#### 4.2.4. Agrochemicals and Reuse of Treated Wastewater

Current agricultural practices include widespread use of agrochemicals, pesticides, fertilizers, and treated wastewater that result in the global exposure of agricultural crops and natural ecosystems to many synthetic chemicals and nanomaterials [128]. Some of these chemicals may impact human health, physiology, reproduction, and development through a variety of neuroendocrine effects [129]. Green leaf biomass, whether produced in conventional, greenhouse, or vertical farming settings, remains at risk of direct exposure and the transfer of these chemicals when integrated into the food manufacturing processes. Precise management of environmental and health risks, sustainable and safe use of agrochemicals, internationally adopted maximum residue limits, and the integration of alternative agricultural management strategies will ensure that consumers can be confident that their food meets the agreed standards for safety and quality.

## 5. Conclusions

Green leaf proteins are ample, versatile, and functional ingredients underutilized in modern agricultural systems. With their abundance, reaching price parity and finding novel technological solutions for low-cost processing of large volumes of green biomass are critical for success in the alternative protein marketplace. Beyond direct incorporation in human foods, green leaf proteins hold an additional promise in the premium ingredient markets that prioritize functionality, such as protein hydrolysates, mixtures of bioactive peptides, fractions with defined enzymatic activities, edible protein films, binders, and coatings. Nutritional and functional profiles of green leaf proteins vary depending on the agricultural commodity and plant source of the protein, which allows for a more precise targeting of niche markets for dietary ingredients and personalized nutritional interventions. As the market for green leaf proteins continues to grow, it will play a significant role in promoting sustainable agriculture, providing nutritious food options, and supporting overall health and wellness.

## Figures and Tables

**Table 1:** Protein content, digestibility, and essential amino acid profiles of major conventional and alternative protein sources.

	Protein Quality						Essential Amino Acid Composition (% Protein)													
Protein Type	100 g Fresh Weight	%Digestibility	%Biological Value	%Net utilization	% PDC AAS	% DI AAS	Arg *	Cys *	His **	Ile **	Leu **	Lys **	Met **	Phe **	Tre **	Trp **	Tyr *	Val **	Met + Cys	Phe + Tyr
Animal sources																				
Beef	22.7	92%	80%	73%	92%	112%	6.3	1.4	2.7	4.9	8.5	8.3	2.4	3.8	4.0	1.3	3.0	5.3	3.8	6.8
Pork	16.9	98%	-	-	70%	-	6.6	1.3	2.8	4.8	7.0	7.2	2.4	4.1	5.0	1.3	2.8	4.2	3.7	7.0
Chicken	20.8	95%	79%	80%	91%	108%	7.0	1.2	4.7	3.8	12.2	5.6	6.8	4.7	3.3	7.4	3.8	5.5	8.1	8.7
Fish ^a^	17.8	94%	67%	64%	106%	100%	4.5	2.0	2.3	3.1	4.7	6.2	3.6	5.5	3.4	0.9	5.7	3.8	5.6	11.2
Eggs ^a^	12.6	98%	100%	94%	100%	113%	5.4	2.2	2.3	5.7	8.5	6.9	3.4	5.8	4.6	1.2	3.9	6.4	5.6	9.7
Milk ^a^	3.3	96%	91%	82%	100%	114%	3.33	0.9	3.6	4.0	8.8	7.7	2.9	4.6	4.8	1.4	5.0	4.7	3.7	9.6
Whey PI	0.9	100%	104%	92%	100%	125%	1.8	2.1	1.3	5.6	10.3	9.7	1.7	2.6	7.9	1.9	2.7	5.9	3.8	5.3
Pulse (legume) seeds																				
Soybean ^a^	13.0	97%	73%	66%	100%	100%	6.2	2.1	3.0	5.3	7.1	6.1	2.7	3.9	3.7	7.6	4.1	5.2	4.8	8.0
Soy flour ^a^	37.8	80%	-	-	93%	105%	7.6	1.5	2.6	4.9	7.8	6.4	1.4	5.2	3.6	1.4	3.8	4.7	2.9	9.0
Soy PI ^a^	88.3	98%	74%	61%	100%	98%	7.6	1.2	2.6	4.8	7.7	6.0	1.3	5.2	3.6	1.3	3.7	4.7	2.5	8.9
Pea, yellow	22.3	87%	64%	56%	78%	65%	8.4	1.4	2.5	4.2	7.1	7.2	1.0	4.7	3.8	0.9	3.1	4.8	2.4	7.8
Pea PI	-	99%	65%	-	89%	-	7.4	0.7	2.0	3.8	7.2	5.8	0.7	4.6	3.0	0.7	3.2	4.0	1.4	7.8
Bean, kidney	22.5	64%	-	-	68%	59%	5.7	0.9	2.7	4.5	7.6	5.5	1.2	5.1	3.4	7.5	4.2	5.0	2.1	9.3
Chickpea	20.5	89%	68%	58%	74%	-	14.1	0.6	4.5	5.1	8.8	10.5	1.4	5.1	3.6	1.0	3.6	5.0	2.0	8.7
Fava bean	26.1	95%	-	-	69%	-	9.0	1.2	2.6	4.1	7.1	6.3	0.8	4.0	3.5	0.8	2.7	4.6	2.0	6.7
Lupin	36.2	76%	83%	-	81%	-	11.0	1.5	2.7	4.2	6.9	4.7	0.7	4.0	3.4	0.8	3.6	3.9	2.2	7.6
Cereal seeds																				
Wheat, grain ^a^	9.6	86%	80%	-	42%	54%	2.4	0.7	1.4	3.0	5.0	1.1	0.7	3.7	1.8	0.3	2.4	2.3	1.4	6.1
Barley, grain	12.5	99%	81%	-	61%	51%	6.0	1.5	2.2	3.6	4.6	0.8	0.7	3.6	1.9	0.7	1.6	3.5	2.2	5.2
Oats, grain	13.5	90%	-	-	-	77%	9.7	1.7	3.6	4.4	9.1	3.7	1.7	5.5	4.3	3.6	2.6	6.0	5.4	8.1
Rice, white	6.8	92%	-	-	63%	64%	5.9	0.2	1.6	2.3	5.7	4.7	0.3	3.7	2.3	1.0	2.6	2.7	0.5	6.3
Rice, brown	7.5	79%	-	-	-	-	7.6	1.2	2.6	4.2	8.3	3.8	2.2	5.1	3.7	1.3	3.8	5.8	3.4	8.9
Corn, grain	9.4	-	-	-	60%	48%	1.7	0.3	1.1	1.7	8.8	1.0	1.1	3.4	1.8	0.6	2.7	2.1	1.4	6.1
Corn, distillers	27.1	-	-	-	-	-	3.4	2.0	2.4	3.5	12.0	2.6	1.9	4.6	3.2	0.5	4.1	4.4	3.9	8.7
Sorghum, grain	10.6	-	-	-	20%	29%	4.1	1.7	1.9	3.1	13.0	2.0	1.2	5.0	3.2	1.8	4.0	4.3	2.9	9.0
Oilseed cakes or meals																				
Canola	39.0	-	-	-	-	-	5.9	2.5	2.6	4.0	6.8	5.6	2.0	3.9	4.2	1.2	2.9	4.9	4.5	6.8
Rapeseed	38.3	-	-	-	92%	70%	6.1	2.3	2.6	4.0	6.7	5.5	2.1	3.9	4.4	1.3	3.1	5.1	4.4	7.0
Sunflower	37.7	-	-	-	99%	97%	8.5	1.7	2.5	4.1	6.2	3.5	2.3	4.4	3.6	1.2	2.4	4.9	4	6.8
Hemp	33.4	87%	-	-	48%	-	12.4	1.8	3.0	3.9	6.9	3.9	2.4	4.7	3.8	1.1	3.2	5.1	4.2	7.9
Flax	34.2	-	-	-	-	-	2.81	2.0	2.7	3.7	5.8	3.6	1.0	5.2	3.7	0.5	2.4	4.7	3	7.6
Cotton	45.0	85%	-	-	-	-	11.1	1.6	2.9	3.2	5.9	4.2	1.4	5.1	3.3	1.1	2.9	4.2	3	8.0
Green leaf or forage crops																				
Alfalfa	5.2	76%		-	57%	72%	4.4	1.8	3.0	4.8	6.9	4.8	1.9	3.9	1.7	1.3	3.5	4.1	3.7	7.2
Spinach	2.9	74%		-	51%	-	5.6	1.3	3.0	3.7	7.0	5.5	1.2	4.5	4.0	1.6	6.1	5.0	2.4	10.5
Sugar beet	2.2	72%	-	-	-	-	5.3	0.3	1.7	6.2	6.8	5.8	1.5	6.0	4.2	0.7	3.3	5.6	1.8	9.3
Cabbage	1.0	82%	-	-	57%	-	4.0	2.4	1.4	3.1	4.1	2.1	4.2	2.8	3.4	1.0	2.1	4.6	6.6	4.9
Lettuce	1.1	91%	77%	-	19%	-	6.3	1.3	1.9	4.5	5.9	6.4	1.5	5.3	4.7	6.1	1.9	6.9	2.8	7.3
Sweet potato	2.5	73%	-	92%	70%	-	6.0	3.8	1.4	3.7	8.6	3.6	1.1	7.0	5.0	0.9	4.1	5.7	4.9	11.1
Cassava	1.8	68%	57%	40%	-	-	5.9	3.6	2.2	5.2	10.5	6.2	1.0	5.7	5.1	1.0	3.3	5.3	4.6	9.0
Duckweed	3.0	65%	-	-	45%	75%	6.6	1.2	1.6	3.6	6.6	4.7	1.4	4.4	3.5	1.4	2.8	4.5	2.6	7.2
Grass, orch.	4.0	69%	-	-	-	-	0.9	0.2	0.3	0.8	1.4	0.8	0.3	0.9	0.7	0.2	0.5	1.0	0.3	1.5
Others (nuts, tubers, etc.)																				
Almonds ^a^	21.2	-	-	-	23%	-	9.3	0.2	2.1	2.7	5.8	2.4	0.4	4.5	1.9	0.9	1.2	3.2	0.7	5.4
Peanuts ^a^	25.8	95%	54%	47%	52%	43%	10.6	1.1	2.2	2.9	6.0	3.4	1.0	4.7	0.1	0.8	3.4	3.6	2.1	8.1
Potato	2.1	82%	-	-	82%	-	3.3	0.3	1.4	3.1	6.7	4.8	1.3	4.2	4.1	0.1	3.8	3.7	1.6	8
Brewer’s yeast	48.6	-	-	-	-	-	4.4	0.9	2.0	4.6	6.2	6.3	1.5	3.6	4.4	1.1	2.7	4.9	2.4	6.3
Mushroom, butt.	3.1	-	-	-	-	-	4.1	0.1	1.6	4.6	7.9	8.1	1.4	4.7	5.6	0.1	2.9	5.9	1.4	7.6

PI, protein isolate (typically less allergenic and digested more slowly); (^a^) major 8 food allergens as identified by the FDA; (*) conditionally indispensable amino acids: tyrosine, cysteine, and arginine; (**) indispensable amino acids: valine, tryptophan, threonine, phenylalanine, methionine, lysine, leucine, isoleucine, and histidine; (-) data not available.

## Data Availability

Not applicable.

## Supplementary Materials

The following supporting information can be downloaded at: https://www.mdpi.com/article/10.3390/nu15061327/s1, Supplementary Table S1: Detailed analysis of commonly used strategies to extract proteins from green leaves.
