# Supplementary material for: Current Status and Nutritional Value of Green Leaf Protein
Source: Nutrients. 2023 Mar 8;15(6):1327. doi: 10.3390/nu15061327 (PMC10056349; doi:10.3390/nu15061327)
Supplement: Supplementary file 1 [file nutrients-15-01327-s001.zip › nutrients-2245093-supplementary.pdf]

nutrients

Review

Current status and nutrient value of green leaf protein

Connor Balfany [1,2], Janelle Gutierrez [1], Marvin Moncada [1,2], and Slavko Komarnytsky [1,2*]

Supplementary table 1. Detailed analysis of commonly used strategies to extract proteins from green leaves.

Protein release	Protein recovery	Plant source (protein content %DM)	Purity of extract %	Extraction efficiency	Treatment release	Treatment recovery	Comments
Alkaline solubilization	Filtration	Saltbush leaf *Atriplex lampa* (22.0%)	85.0%	Not disclosed	Mechanical grinding of fresh leaves done at 5C, with juice being adjusted for pH, water content, and holding time. Centrifuged to remove crude material.	Pressed juice was chilled and passed through a continuous recirculating membrane filter system of 10 kDa. Pressure was adjusted based on fouling and feed rate. Diafiltration was performed by diluting the retentate with treated water.	Salt reduction of the halophytic plant was over 90%, making this a good option to remove anti nutritive compounds that could interfere with protein digestibility. Biological value of the final protein product was 79.8 compared to caseins at 91.6.
Alkaline solubilization	Acid IEP, ammonium sulfate precipitation, combination of both	Green tea leaves *Camillia sinensis* (ND)	89.7%	29.7%	0.5 M NaOH added in different temperature, pH, time of contact, and ratio variables. Supernatant saved for recovery.	1 N HCl IP with variable pH ranges 1-7; 40-90% concentration ammonium sulfate, acidification to pH 5 and 80% ammonium sulfate. All centrifuged.	Increasing the treatment time of alkaline solubilization to 100 min quadrupled yield from 20 min treatment. 80C residency, 1:40 solid to liquid ratio and pH exceeding 12 showed increases in extraction.
Alkaline solubilization and enzyme extraction	IEP, ammonium sulfate, IEP and ammonium sulfate	Sugar beet leaf *Beta vulgaris* (24.1%)	69.08%	79%	Sugar beet leaf was blended at a 1:10 (w/v) ratio at pH 6 in sodium acetate buffer. Suspension held at 45C for 30 min, pH adjusted to 8.5 with 1M NaOH, and filtered to remove fiber. Enzymes introduced at 8% concentration during 45C hold.	Filtrate was adjusted to pH 4.5 with 1M HCl and/or ammonium sulfate at 85% concentration. Centrifugation of concentrated protein pellet, dialysis performed on sulfate treated samples. Samples freeze dried.	High solubility at pH 7.5 (98.7%), low bulk density (80.7 kg/m3), and dark color due to chlorophyll degradation. Good flowability (19.1%) and intermediate cohesiveness (1.2%). Dispersibility of 77.9%, good composition of essential amino acids.
Alkaline solubilization and enzymatic digestion	Ammonia sulfate precipitation	Green tea leaf *Camellia sinesis* (21-28%)	ND	56.4%	Green tea leaves covered with boiling water, dried, mashed and put through a sieve. Mashed leaves either treated with NaOH solution, or treated with enzymes (neutrase, alcalase, protamex, flavourzyme).	Supernatant was precipitated with the addition of ammonia sulfate, and collected through centrifugation. Protein pellet was mixed with acetone at 1:1 ratio, stirred for 30 minutes, then filtered. Acetone was repeated 4 times. Dialysis done for 2 hours on final washed pellet.	Alkaline extraction method outperformed the enzyme treatments (56.4% vs. 47.8%). Of the enzymes, alcalase and protamex combination was the most effective.

Mechanical juice expression (twin screw press)	Acid IEP	Sugar beet leaves (19.4%)	34.5%	65%	Sugar Beet Leaves juiced with Angel juicer; juice immediately went to treatment.	1 N HCl IP to pH 3.5 and 4.5, stirred for 1 hour, centrifuged, pellet collected and freeze dried.	Separating soluble from insoluble proteins reduced extraction efficiency from 65% down to 8%. Washes with acetone removed color but only increased protein purity to 42%.
Mechanical juice expression	Heat coagulation	Alfalfa *Medicago sativa* (ND)	50-61%	ND	Fresh alfalfa in late bud stage was wet fractionated, but methods not mentioned. Juice was retained for protein extraction	Alfalfa juice was subject to steam injection at 60C or 80C for 30, 60, 120, 240, or 480 min. Coagulate recovered through cheese cloth and freeze dried.	More protein recovery in the 80C treatment, however longer residency times (480 min) decreased recovery, indicating protein breakdown. Functional properties such as solubility were worse in 80C fraction.
Mechanical blending with water or juice expression	Heat, acid IEP, or heat and filtration	Duckweed *Lemna gibba* (33.6%)	67.2%	14.2%	Fresh duckweed was either blended with DI water, supernatant saved or passed through screw press to create a juice.	Juice was either heat treated at 70C for 1 hour, precipitated with acid (HCl), or heat treated between 50-54C at pH 6 for 20-60 minutes, centrifuged to remove green protein/chlorophyll, and supernatant filtered (cross flow filtration to ultrafiltration to diafiltration). Filtered protein freeze dried.	No functional or proximate tests done on heat or IEP recovered pellets, only on filtered protein. Originally alkaline juice expression was used, but was abandoned because it removed functionality. Additionally heating at pH 6 was used because of higher protein recovery than pH 6.5-7.
Mechanical pressing and juice expression	Acid IEP, heat	Alfalfa *Medicago sativa* (20.7%)	43-46% (green) 65-73% (white)	15-18% (green) 4-6% (white)	Fresh alfalfa was harvested in prebloom, and juiced with a screw press, yielding 53% of weight in green juice	Two flocculant treatments (Magnafloc LT-27, Gigtar G-5) were added at 0.03%. Sediment removed through filtering. Brown juice subjected to heating (85C) or IP (pH 3.5, 2N HCl) and centrifuged to recover green fraction.	Addition of flocculants helped remove chloroplastic sediment. High alkalinity of the juice helped increase effectiveness of the flocculants. Alkali range of 7.5 was also shown to preserve xanthophyll decomposition and reduce proteolytic enzyme activity.
Mechanical pressing and juice expression	Acid (lactic, citric), fermentation (*L. salivarius,* endogenous)	Alfalfa *Medicago sativa* (24.5%)	41-53%	51-76%	Fresh alfalfa was harvested as flowering began, stored at -20C till use. Juiced with screw press.	Four fractions made.1. 1mol/L citric acid 2. 3 mol/L lactic acid. 1 and 2 adjusted to pH 4.5. Stored at 4C for 9h. 3. Juice inoculated with 5% w/v MRS broth, incubated 4C and 38C. Centrifuged to remove pellet.	Fermentation destroyed the subunits of rubisco and is evident of proteolytic activity, acid precipitation preserved. Higher preservation of essential amino acid cysteine in acid recovery.
Enzymatic digestion	Acid IEP	*Moringa oleifera* leaf powder (23.6%)	55.6%	14.2%	Fresh moringa leaves were harvested, air dried, and ground. Flour was defatted with hexane. Viscozyme L was added to the flour while varying abiotic factors.	Supernatant is acidified to 4.5 with 2N HCl and left overnight at 4C. Centrifuged to remove precipitated proteins, which then underwent freeze drying.	After enzymatic digestion, solution brought to pH 11 with 2N NaOH and centrifuged to remove fiber. Amino acid score remained fairly consistent between leaf and concentrate, but protein digestibility went from 64.7% to 99.8%.

Enzymatic digestion	Acetone precipitation	Olive leaf *Olea europeaea* (5% by fresh weight)	N/D	N/D	Fresh leaves frozen in liquid nitrogen and powdered. Mixed with water, enzyme (Cellulclast 1.5L), and adjusted to pH 5.0 with sonication. Centrifuged to remove fiber.	Supernatant was precipitated by addition of acetone. Proteins were collected through centrifugation.	Optimal variables for enzyme digestion increased extraction yield by 90% compared to solubilization/precipitation that did not use enzymes.
Enzyme digestion, alkaline, ultrasonic, mechanical	Acid IEP	Jackfruit leaves *Atrocarpus heterophyllus* (13-16%)	41.4%	7.0%	Jackfruit leaves picked fresh, dehydrated, and ground using a blender. Flour was mixed with acetone to depigment, air dried and mixed with water and 0.2M NaOH.	The three aliquot treatments were: control, subject to ultrasonic bath for 20 min, or treated with pancreatin (1% w/v for 180 min).	Extensive functionality testing was done on protein fractions, all were deemed undesirable. Enzymatic extraction improved functionality and antioxidant properties. Ultrasonic treatment enhanced enzyme hydrolysis.

DM, dry matter; ND, not determined.
